# Edible Oil Parameters during Deterioration Processes

**DOI:** 10.1155/2021/7105170

**Published:** 2021-09-17

**Authors:** Marcos Flores, Victoria Avendaño, Jessica Bravo, Cristian Valdés, Oscar Forero-Doria, Vilma Quitral, Yesica Vilcanqui, Jaime Ortiz-Viedma

**Affiliations:** ^1^Departamento de Ciencias Básicas, Facultad de Ciencias, Universidad Santo Tomás, Avenida Carlos Schorr 255, Talca, Chile; ^2^Facultad de Medicina, Centro de Investigación Biomédica, Universidad Diego Portales, Ejército 141, Santiago, Chile; ^3^Centro de Investigación de Estudios Avanzados del Maule, Vicerrectoría de Investigación y Postgrado, Universidad Católica del Maule, Talca, Chile; ^4^Escuela de Nutrición y Dietética, Facultad de Salud, Universidad Santo Tomás, Ejercito 146, Santiago, Chile; ^5^Escuela de Ingeniería Agroindustrial, Universidad Nacional de Moquegua, Prolongación Calle Ancash S/N, Moquegua, Peru; ^6^Departamento de Ciencia de los Alimentos y Tecnología Química, Facultad de Ciencias Químicas y Farmacéuticas, Universidad de Chile, Casilla 233, Santiago, Chile

## Abstract

With the continuous increase in research on lipids, technologies and the development of chemical-analytical methods associated with the characterization and monitoring of different processes that involve modifications in edible fats are increasing. The beneficial effect of lipids, especially those essential for the health of the population, is widely known. However, degradation compounds are also produced that eventually have negative effects. In this dual context, the monitoring of the changes suffered by nutritional compounds can be obtained thanks to the development of technologies and analytical methods applied to the study of lipids. The modifications that lipids undergo can be followed by a wide variety of methods, ranging from the basic ones associated with simple chemical titrations to the more complex ones associated with sophisticated laboratory equipment. These determinations involve chemical and/or physical quantification of lipids to know an initial condition on the major and minor components. In addition to technologies that allow monitoring during more complex processes such as thermal deterioration, in multiple conditions depending on the objective of the study, this review could benefit a comprehensive understanding of lipid deterioration for future developments and research in the study of fats and oils for human consumption.

## 1. Introduction

Lipids are a group of macromolecules of biological importance for humans, since they have several functions in our metabolism: storing energy, being part of cell membranes, functioning as hormones, and giving food texture and flavor [1]. They can be in solid form (fats) or in liquid form (oils). The oils are composed of triacylglycerols (TAG) that determine their physicochemical characteristics.

Fatty acids are the simplest lipids, chemically defined as a nonpolar, linear, unbranched hydrocarbon chain, which may be saturated or unsaturated, with a carboxyl group at the end. The linear chain has a definite beginning and end; therefore, it has no rings [2].

Edible oils, before being ingested, can be used at high temperatures as a sauteed or during the frying process; in this way, the surface of foods that have a protein origin or that have a high carbohydrate content is modified. In the frying process, the chemical nature of the oil deteriorates, especially if there are unsaturations, due to the formation of compounds that could be toxic when its consumption is moderate, and very harmful to the health of man when it is chronic, added to the generation of other volatile compounds when the oil is in contact with food at high temperatures [2–4]. For this reason, the fact of following up during the process of deterioration of a particular material or chemical compound becomes relevant.

Also, when the oils are in contact with air and humidity, they undergo a process called autooxidation that as a result shows the appearance of odors, flavors, and abnormal colors, formation of possibly toxic compounds and the decrease in the usefulness of the product. This process is divided into three phases: initiation, propagation, and termination. So that the oils can have a longer useful life and generate good quality products when subjected to heating, antioxidants are added, ideally a mixture of them, since they have a synergistic effect and generate greater protection. The most commonly used are tocopherols, terbutyl hydroquinone (TBHQ), butyl hydroxyanisole (BHA)-TBHQ, and citric acid mixture [5].

As described above, lipid modifications can be quantified and characterized by static and dynamic methods that determine their oxidation against different environmental conditions. Due to this, the objective of this review is to review these methods, describe and compare them, to generate new knowledge and alternatives to improve the study of vegetable oils during heating processes.

## 2. Methods for Evaluating the Stability of Edible Oils

### 2.1. Static Methods

They are methods that evaluate one or several functional groups that may or may not be present in the thermooxidation process, thus giving an assessment of the in situ state of the oxidation of a fat.

#### 2.1.1. Initial Composition of Fatty Acids

Fatty acids are organic components of lipids that for man fulfill an energy reserve function and help tissue development. Depending on the amount of double bonds (C = C) that present or not, they are classified as saturated and unsaturated. Saturated fatty acids generally have an animal origin, except for coconut and cocoa oil, which are solid at room temperature. From the cardiovascular point of view, the risk of developing a cardiovascular disease (CVD) and increasing blood cholesterol levels increases. On the contrary, unsaturated fatty acids such us monounsaturated (MUFA) and/or polyunsaturated fatty acids (PUFAs) have, in general, a plant origin, they are liquid at room temperature, and for cardiovascular health, they are beneficial for increasing HDL cholesterol [1].

However, PUFAs are abundant in edible vegetable oils; being the main substrates for the process of oxidation of oils, the products generated deteriorate the chemical, sensory, and nutritional properties of oils. In this sense, it is useful to determine the fatty acid composition of an oil, and one of the most used methods is the analysis of methyl esterified fatty acids (FAME) by gas-liquid chromatography (GC), which in addition to providing the percentage of ester in the fluid, provides the percentage of individual esters according to the structure of fatty acids [6].

The EN 14103 method is widely used commercially, capable of determining the percentage of FAME by gas phase chromatography using methyl heptadecanoate [7]. The results corroborate that it provides a robust analysis regarding the components of the oil, in addition to its high replicability in edible oils [8]. Despite the good figures of merit that can be obtained by chromatographic methods, it should be considered that the analysis is carried out on the modified compound of interest; the chromatographic methods are time consuming and results depend on detector type.

On the other hand, oils for food use, besides presenting fatty acids, have a large amount of protective or bioactive components such as phytosterols, phytoestrogens, flavonols, carotenes, and tocopherols, which are described as antioxidants because they help prevent diseases and have an important nutritional contribution [9]. Also, the lack of antioxidants in human metabolism facilitates the development of degenerative, cardiovascular, and nervous system diseases; an alternative to improve the components of the diet and therefore the health of the population is the addition of antioxidants of plant origin in the production of different types of food [10].

#### 2.1.2. Primary and Secondary Oxidation Products

One of the phenomena that oils present is self-oxidation, rancidity, or rancidity, which is defined as a natural process that occurs between fats and oils with oxygen and moisture, suffering a change in their chemical characteristics, smell and taste. There are two types of autooxidation: oxidative and enzymatic. The first, results in the appearance of odors, flavors, strange colors, toxic compounds, and the decrease of the product's useful life. In addition, it destroys fat-soluble vitamins vital for human functioning such as vitamin A and E. It is divided into three stages [5].

- Initiation: the existence of unsaturated fatty acids allows hydrogen adjacent to the double bond to form free radicals, which end up accumulating; on the other hand, partial reduction generates “reactive oxygen species” (ROS) such as hydrogen hydroperoxide (H₂O₂) and free radicals: superoxide (O₂¯), hydroperoxyl (HO₂), and hydroxyls (OH). The compounds mentioned above, in humans in moderate concentrations, fulfill a physiological energy role, but in high concentrations due to diets rich in fats and oils generate oxidative stress to the body's cells [11]. The initiation phase is dependent on oxygen and/or heavy metals such as iron, cobalt, manganese, in light and high temperature
- Propagation: accumulated free radicals cause peroxide radicals that in turn give rise to hydroperoxides (primary oxidation compounds). These are capable of modifying enzymes, injuring the digestive system and can be mutagenic
- Termination: the existence of free radicals generates dimers, which causes the previous phase to stop and gives rise to volatile/nonvolatile compounds, responsible for the chemical changes mentioned above; most are aldehydes that are detected quickly through smell

On the other hand, the second type of oxidation is due to the performance of two types of enzymes: lipooxidases and peroxidases, which catalyze the formation of peroxides. They give rise to characteristic smells and flavors.

However, one of the problems in the area of health is that the autooxidation and heating process generate primary products such as peroxides and secondary products such as aldehydes, ketones, alcohols, and polymers that possess a cytotoxic action.

Grompone determined that oil may have a low peroxide index due to its good quality or the treatment to which it was subjected, but this does not guarantee that it has good stability during storage [12].

The peroxide index has been widely used in lipid oxidation, although it is only limited to the initial stages of this process. As peroxides undergo subsequent decomposition reactions, it is necessary to use the anisidine index or the thiobarbituric acid index that can measure secondary oxidation products and also reveal the complete history of the analyzed oil [12]. In the presence of acetic acid, p-anisidine reacts with aldehydes producing a yellow color, and, in addition, the p-anisidine value is defined as 100 times the optical density measured at 350 nm in a 1 cm cuvette of a solution that has 1 g of oil in 100 ml of solution.

**Figure 1:**
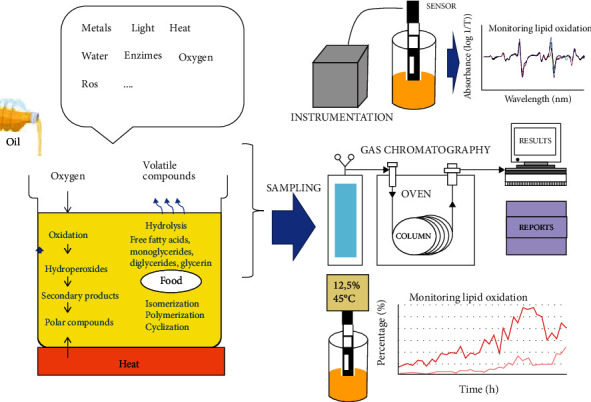
Diagram of the process of obtaining information during the deterioration of edible lipids.

As the two indices mentioned above are able to evaluate in a complementary way lipid oxidation, the so-called TOTOX index or oxidation index (OV) was proposed, which is equivalent to 2x peroxide value+p-anisidine value.  Figure 1 shows a diagram of the process involved during the deterioration of edible lipids.

#### 2.1.3. Chemical Measures of Oils

*(1) Acid Value*. Another of the concepts related to the process of lipid oxidation is the oil acidity, which is a measure of the degree of decomposition of the oil by the action of lipases or other causes. This process is accelerated by light and heat. The acidity index of an oil or fat is defined as the number of milligrams of potassium hydroxide (KOH) required to neutralize free acidity per gram of sample [6, 13]. This value of the degree of acidity is expressed referring to the percentage of oleic acid. In some cases, it may be referred to palmitic acid, lauric acid, or others [6, 14].

García et al. developed a study of the hydrolytic deterioration of olive oil by analyzing the free acidity that olive oil presents, establishing that the measure of acidity is one of the chemical characteristics that best define the quality of an oil olive, because it gives an idea of how the olive has been cultivated, collected, stored, and transported, as well as the production of oil in the mill [15].

*(2) Iodine Value*. It is considered one of the oldest and most traditional methods to determine the unsaturation (double bonds) of the oil or ester [6]. It can be determined by dissolving a sample of oil (previously mass) in a nonpolar solvent such as cyclohexane that reacts with glacial acetic acid in a redox reaction. This reaction is carried out in dark conditions to prevent lateral reactions of light-induced radicals that could affect the result [16]. It is expressed in a conventional manner by the weight of iodine absorbed by one hundred parts by weight of the fat [6, 17].

To determine this index, the Wijs method is the most used, the lipid must react with iodine monochloride solution, in a mixture of acetic acid and carbon tetrachloride, releasing the excess iodine and titrating it with sodium thiosulfate.

Edible oils, in general, have a large amount of unsaturated fatty acids, so their iodine levels will be higher at higher unsaturation. There is a directly proportional relationship between the degree of unsaturation and lipid oxidation.

The iodine value has been used as a reference method for the development of new methods for the study of the thermal degradation of edible oils. Lam et al. propose a new robust method based on surface enhanced Raman spectroscopy [18]. The change in the ratio of the vibrational signals from unsaturated fats (1265 cm¯^1^) to saturated (1440 cm¯^1^) with the application of the heat treatment basically allowed to study the thermal deterioration [18]. This trend was related to the changes in the iodine value.

*(3) Saponification Value (SV)*. It is an approximate measure of the average molecular weight of the fat studied and denotes the reaction that occurs between a strong base and the fat, giving a product a soap (alkaline salt of fatty acids) and glycerin [13]. This is expressed as the weight in milligrams of potassium hydroxide needed to saponify a gram of fat [6, 14].

Fats that have short-chain fatty acids consume more KOH, so they have higher SV while those that have long-chain fatty acids consume less alkali showing lower SV, as is the case with vegetable oils such as sunflower seed oil or soybean oil that have an SV of 191-194, while lauric oils such as coconut oil and palm oil have 255-270 mg KOH SV per gram of oil. Due to this, its use has been an essential tool in the characterization of vegeta-ble oils [19].

*(4) Thiobarbituric Acid Index*. It is one of the oldest and most used tests to evaluate the oxidation of lipids in foods and other biological systems, being very useful to compare samples of a certain oil in the different stages of the oxidative process. It is expressed as milligrams of malonaldehyde (MA) equivalents per kilogram of sample, or as micromoles of equivalents of MA per gram of sample. MA is a product that is generated by the oxidation of polyunsaturated fatty acids that, when reacted with the thiobarbituric acid (TBA) reagent, forms a pink complex with a maximum absorption between 530 and 532 nm [20]. In general, significant amounts of compounds that react with TBA are produced only when it comes to fatty acids with three or more double bonds.

Shen et al. used the TBARS index to monitor the protection that 4 different types of antioxidants with various antioxidant mechanisms can exert on docosahexaenoic acid (DHA) algae oil, concluding that an optimum antioxidant composite allows to extend the useful life in 3.8 times more than the control sample [21].  Table 1 shows the advantages and disadvantages of the basic methods of determination of primary and secondary compounds.

#### 2.1.4. Physical Measures of Oils

*(1) Density*. Density is a physical quantity that measures the mass per unit volume of an oil or fat. This value decreases linearly with increasing temperature, that is, in an inversely proportional relationship [27]. On the other hand, at a given temperature, the higher the molecular weight of the oil, the greater its density [28]. For its determination, the temperature must be exactly controlled because the density of fatty substances varies approximately 6.8 × 10^4^ g/ml per degree and a pycnometer is used. For fats and liquid oils, this instrument must be at a constant temperature of the environment, while for solid fats, it must be at the melting temperature of the studied fat [14].

*(2) Viscosity*. Viscosity (*η*) is the measure of the internal resistance of an oil against the free flow of a liquid to the frictional forces between the layers of fluids that move over each other at different speeds. It is considered an important property that allows vegetable oils to be used in the preparation or processing of food in homes, industries, or restaurants. While it has a greater amount of polyunsaturated fatty acids, its viscosity is low, and therefore, it is not useful for some food processes [1]. It is physically influenced by different parameters such as temperature, density, and pressure (*η* = *f* (*T*, *ρ* *p*)).

From an economic point of view and optimization of the use of time, mathematical models have been proposed based on the parameters mentioned above, in order to determine the viscosity of the oil in a more exact way [29].

*(3) Viscofrit*. It is a test that allows measuring the time required in seconds for a sample of frying oil (liquid) to leave a funnel, at a temperature range of 15 to 50°C [30]. It does not use chemical reagents, electricity, or any consumable material, and the maintenance cost is zero.

This system consists of a small cone of standard measurements that incorporates a thermometer with temperature and time scales. It is calibrated, so that if the emptying time of the cone exceeds the time indicated on the thermometer, it means that the oil contains a level of polar and polymer higher than the 25% limit set by law [31].

One of the advantages is that this system incorporates different thermometers that are interchangeable and that are calibrated for the different oils that can be used in the frying.

Osawa et al. evaluated the effectiveness of the Viscofrit rapid test in the evaluation of oils used in the frying process, concluding that it is an effective system to measure the amount of polar compounds in the oil but is not recommended for oils that have high points of fusion [30].

*(4) Refractive Index (IR)*. It is defined as the relationship of the speed of light in a vacuum with the speed of light in oil at a defined temperature. It also gives a measure of the purity and quality of oils both at the laboratory and industrial levels. This index is measured with a refractometer, generally between 20 and 25°C for oils and 40°C for solid fats (liquefied). This physical parameter decreases linearly (directly proportionally) along with the iodine index; and both indices have an inverse relationship with temperature, that is, when the temperature increases these two indices decrease; therefore, it is therefore also used as an index to report the degree of hydrogenation of the oil [20].

Paucar-Menacho et al. conducted a comparative study of the physical chemical characteristics of sacha inchi oil, olive oil, and raw fish oil in order to determine the potential health benefits; one of the parameters measured was the refractive index; concluding that at higher IR of the studied oil, the degree of unsaturation is higher (directly proportional ratio), sacha inchi oil presented a higher IR than the other oils, and this oil is highly unsaturated (91%) which makes it advisable to human consumption, as it is related to effects on weight control and obesity [32].

*(5) Melting Point*. Both fats and edible oils, being mixtures of glycerols and other substances, do not have a net and definite melting point, neither does it present a critical point of the passage from solid to liquid, since this process is a gradual transition through different states to reach the end. For this reason, the melting point of grease is defined by two temperatures: the initial sliding softener and the final perfectly clear liquid. This measure decreases if the fatty acid is unsaturated and fluidity increases, because they do not have a crystalline structure but rather have breaks in the linear structure [33].

To corroborate the fact that the fusion of an oil is a gradual process, Fasina et al. studied 12 vegetable oils which were analyzed experimentally: almond oil, canola, corn, grapes, hazelnut, olive, peanut, safflower, sesame, soy, sunflower, and walnut oil within a temperature range of -60 to 25°C by means of differential scanning calorimetry (DSC) [34]. The results showed that the samples of vegetable oils melted in a large temperature range (19-44°C) and not only at a temperature; this is because they have a different triacylglycerol composition, finding a high correlation between the amount of mono/unsaturations of the oil with the starting melting temperature, maximum melting, and the enthalpy of melting for the 12 samples. In addition, the melting characteristics of the respective vegetable oils were linearly correlated (*y* = *mx* + *b*) with the amount of monounsaturated or polyunsaturated fatty acid, which is of utmost importance to predict the behavior of an oil and model processing operations of vegetable oils [34].

*(6) Infrared (IR) Spectroscopy*. The spectrum of oil provides important information about the structure and functional groups of the lipid and also the impurities associated with it. It can be used to obtain qualitative data information of the sample studied.

Precisely, infrared spectroscopy, also known as FTIR (Fourier transform infrared) allows to study the phenomena of interaction between infrared radiation and oil. The irradiated energy is in a certain infrared wavelength, which is absorbed by the oil molecules that are vibrating in their basal state at the same wavelength as infrared radiation, generating a change in the level of vibration [35].

In the IR spectrum of oils, there are a number of particular bands, with small variations between the lipid samples, for example, the olefinic bond (= CH at 3010 cm¯^1^), methyl and methylene (CH₃ and CH₂ at 2950, 2850, 1470 cm¯^1^), esterified carbonyl (COOR at 1750 cm¯^1^), free acid at 1710 cm¯^1^), *trans*-bonds (965,941 cm¯^1^), and *cis*-bonds (1460 cm¯^1^) [36]. Due to this valuable information, it has been used to measure the oxidation of oils and recognize their functional groups and *trans*-double fatty acids. It is considered to be fast and simple and requires small amounts of the lipid sample, approximately 20 mg [20].

The IR spectrum is obtained from an infrared spectrometer; basically, its operation consists of infrared radiation that comes from the source that is divided into the divider and one part goes to the moving mirror and another part to the fixed mirror. Then, these two rays are recombined to generate the spectrum, which graphically represents the IR absorbance and the wavelength at which this phenomenon occurs [35].

Factors that can influence IR spectra [35]

1. Representative sample of the oil that contains the complete chemical composition of the oil to be studied or a large part of it
2. Solvents such as water, ethanol, and methanol can cover spectral signals that can interfere with the result of the analysis
3. In some cases, it is better to extract a portion of the total sample that is not harmed by other compounds to obtain a clear and objective spectrum.

#### 2.1.5. Total Polar Compound Value (TPC)

In the frying process, several compounds with a higher polarity than the original triacylglycerols are generated. These compounds include monoacylglycerols, diacylglycerols, and free fatty acids present in unheated fats and products transformed in the heating process; those that are not volatile and increase as the frying process progresses [25].

This index offers the best indicator of the degradation of the oil subjected to frying since it directly measures all the products that degraded in the oil and is applicable for all oils and fats, both animal and vegetable. Because of this, some European countries established a maximum permissible level for TPC in the frying of oil for sanitary regulation. However, it is not clear because there are differences in the level of TPC discard between European countries, for example, Austria 27%; Belgium, France, and Spain at 25%; while Germany does not allow more than 24% [37].

According to the laws or regulations of frying oil, the maximum content of TPC has been set at 24% in Germany; 25% in Belgium, France, Portugal, Italy, and Spain; and 27% in Australia, China, and Switzerland, respectively [38]. In countries such as Canada and the United States, there is still no specific rule for the limit of this value.

The principle used is to determine the total polar compounds by calculating the difference between the weight of the sample added to the chromatographic column and the eluted nonpolar fraction. The official methods for determining the TPC index involve a gravimetric technique that uses chromatographic separation of polar and nonpolar components on a silica gel with water set at 5% [39]. The TPC are the remaining components that remain in the column after elution and is expressed as a percentage of the initial sample weight [37]. Tests conducted by the International Union of Pure and Applied Chemistry (IUPAC) and American Oil Chemist's Society (AOCS) demonstrate that this method is accurate and reproducible with a coefficient of variation of less than 5% [6, 40, 41].

#### 2.1.6. Food Oil Monitor 310 (FOM 310)

It is a direct measuring device that allows to measure the quality of the oil through the percentage of total polar compounds and the dielectric constant they present [42]. It presents a sensor that is submerged in oil subjected to the frying process for one minute, determining if the oil can continue to be used or not.

#### 2.1.7. Testo 270

It is a team that is based on changes in the dielectric constant of the oil that is subjected to high temperatures, being able to evaluate its quality. Its operation is similar to FOM 310.

Cascant et al. studied the effectiveness of Testo 270 to evaluate the oil subjected to a frying process, obtaining high reliability of the results when compared to other rapid tests such as Fritest and has few false negative results [43]. On the other hand, it is recommended that the device be calibrated with oil known to the researcher, in order to minimize reading errors. Even the technology applied by Testo has been used for the development of new sensors related to the determination of polar compounds in a period of oil heating, based on the measuring changes on its electrical capacitance [44].

### 2.2. Dynamic Methods

They are those that force the oxidation of fats by measuring their evolution in a certain time and variables.

#### 2.2.1. Rancimat Method

It has been available since the 80s, being able to determine the oxidative stability index of oil; its great limitation with respect to other methods is that it is only able to run eight samples at the same time. This method consists in subjecting a sample of oil to a forced oxidation by means of dry and filtered air flow at a defined temperature, which, after a certain induction period, emits volatile degradation components, such as formic acid using an automated final detection point [45]. Then, these components are transported by the air stream to a container containing distilled water and the conductivity is measured. The appearance of volatile compounds is recorded as an increase in conductivity. With this method, oils can be classified according to their induction period, which is the time that elapses until the appearance of volatile compounds, the longer the induction period, the more stable a sample is, becoming a standard parameter of quality control of the production of an oil, in addition to being able to estimate the useful life of fatty substances [46].

Due to the aforementioned characteristics, it is widely used because it is reliable and reproducible, does not require reagent consumption, and its measurements can be automatically monitored over time, in addition to including its use as a reference method for the development of other methods with other techniques for the determination of oxidative stability of oils [47, 48].

#### 2.2.2. Active Oxygen Method

Also known as the Swift test of the American Oil Chemists' Society, it is one of the most common methods to assess the oxidative stability of edible fats and oils, used for 60 years with different modifications. It is based on the principle that the aging and rancidification of oil are greatly accelerated by subjecting it to a constant high temperature in a glass tube while bubbling dry air at a certain rate [49]. In spite of the great use that this procedure has had, two important limitations have been identified: the first one is that the end point is determined by the accumulation of the peroxides generated, which are unstable and break down quickly, so that alter the results; and the second is that, during the rapid oxidation phase, the reaction is very susceptible to variations in the oxygen supply, since only the peroxide values of the oil can be determined, while Rancimat allows measuring changes in conductivity caused mainly by polar volatile organic acids, such as formic acid, continuously and automatically [20].

#### 2.2.3. Schaal Test or Schaal Stove Method

It consists of placing an oil sample at 65°C in an oven and aliquots are taken periodically for chemical analysis, until oxidative deterioration is detected. Detection can be performed by measuring organoleptic characteristics or by measuring the peroxide index [50].

Wang et al. evaluated the use of rosemary leaf extracts in the protection of lipids rich in *ω*-3, showing that these extracts have a significant protective effect compared to other natural and synthetic antioxidants, under an oven test condition of schal (60°C) [51].

#### 2.2.4. Microwave

The use of microwaves to heat food is an alternative frequently used in homes as it is fast and uniform. Electromagnetic waves agitate molecules with bipolar moment in food, which can raise the temperature in food. This agitation is a physical mechanism, a simple movement of the molecules to the rhythm of the frequency. Microwave irradiation causes changes in the chemical composition of edible oil during the process of hydrolysis, oxidation, and polymerization reactions which produce rancid flavors and odor in the edible oil [52, 53].

The use of microwaves allows the thermal study of new sources of edible oils as used for heating food in the home [54]. Mazaheri et al. observed that the pretreatment of moistened seeds (8%) with microwaves favors extraction performance, oxidative stability, phenolic content, and other physicochemical properties, probably due to the inactivation of oxidative enzymes and higher content of phenolic compounds [55].  Table 2 shows the advantages and disadvantages of the different accelerated oxidation methods.

### 2.3. Chromatographic Techniques to Characterize Edible Oils

Edible oils, in general, are complex mixtures of compounds of different nature present in liquid or gaseous samples and require greater separation to pure components if necessary. In order to perform a complete analysis, lipids can be separated into their various polarity components (polar and nonpolar fractions) or an analysis of triacylglycerols, free fatty acids, sterols, esters, glycolides, among others [58].

Ghafoor et al. evaluated the behavior of the fatty acid profile of Shia oil subjected to high temperatures, concluding that an oil containing polyunsaturated fatty acids (PUFA) such as Shia oil (PUFA > 80%) should not be heated to a higher temperature at 90°C preserving its nutritional properties [59].

#### 2.3.1. Column Chromatography

It is one of the most used methods for the purification and separation of different organic compounds that are in solid or liquid state.

This segregation occurs given the existence of two effects that are juxtaposed:

- Retention: an effect that occurs on the components of the oil by the stationary phase, which can be solid or liquid anchored to a solid support
- Displacement: effect exerted on the oil components by a mobile phase, which can be liquid or gas

The movement of the different oil compounds through the stationary phase, pushed by the mobile phase, is known as elution. The most used methods in the lipid fractionation of this technique are liquid solid (adsorption), liquid liquid (partition), and ion exchange [60].

In general, column chromatography is based on the fact that the stationary phase is placed in the glass column and the oil is deposited on top of the stationary phase while the mobile phase passes through the system. The different compounds are coming out separately and remain in fractions; the more polar ones are more retained, and for them to come out, it is necessary to change the polarity of the solvent [61].

In adsorption chromatography, the compounds bind to the solid by polar, ionic, and nonpolar forces or van der Waals forces. Therefore, the separation of lipid components occurs thanks to the relative polarities of the individual components, which depend on the number and type of nonpolar hydrophobic groups. These components are generally ordered in the following order: saturated hydrocarbons, unsaturated hydrocarbons, waxes, long chain aldehydes, free fatty acids, quinones, and sterols [20].

#### 2.3.2. Gas Chromatography

A method that consists of dividing the components of the lipid mixture in a vapor state between a mobile gas phase and a nonvolatile stationary immiscible liquid phase dispersed in an inert support. This technique can be used in the analysis of fatty acid composition, which, after transmethylation, are separated by gas chromatography (GC) and detected mainly by flame ionization detection (FID). The recognition of chromatographic peaks is based on the comparison of retention times between samples. In addition, when food lipid triacylglycerols are analyzed, it can provide information about the positional distribution of fatty acids [20].

For the gas phase, it is recommended to use inert gases such as helium that achieves faster separations; however, it is more expensive than the use of nitrogen. On the other hand, hydrogen could also be used because of its rapidity in the method, but it is extremely flammable and requires special handling care. On the other hand, the liquid phase, in general, is a polymeric structure with high stability and viscosity to be in the column at the required temperature, which can dissolve solutes quickly [62].

The incursion of GC in the field of fats and oils has had great results in both animal and vegetable lipids, allowing the analysis of the composition of fatty acids, triacylglycerols, sterols, and other minor components [63]; in this way, lipid analysis has been facilitated because it is fast, reproducible, and quantitative, allowing to distinguish isomers of fatty acids and *trans*-fatty acids [64].

Salimon et al. applied the GC technique coupled to FID for the separation, identification, and quantification of *cis-* and *trans*-fatty acids in edible oils, coinciding with Peng et al., in that it is an effective tool for the study of edible oils [65, 66].

Although complex mixtures of volatilized compounds can be analyzed by GC, the coupling of this method to mass spectrometry (GC-MS) provides additional structural information useful for assessing oil quality. The identification of volatile compounds by GC-MS formed when frying food represents an important tool to identify specific compounds of thermal degradation of oil and can help to identify different frying conditions and elucidate the chemical reactions that occur during the frying process [67, 68]. In this sense, the compounds (E, E)-2,4-decadienal, and (E)-2-undecenal have shown good correlations with the TPC values during frying processes [67].

On the other hand, Peng et al. developed a study to detect and quantify the amount of sesame oil present in a mixture of vegetable oils by GC, being able to obtain the proportion of each component, concluding that it is a very useful method for this type of studies and thus evaluate the quality of oil [66].

#### 2.3.3. Two-Dimensional Gas Chromatography or GCxGC

In the area of edible oils, GCxGC increases the separation potential and sensitivity compared to one-dimensional GC and offers an attractive alternative in FAME analysis. This method achieved excellent separation of geometric and positional isomers of unsaturated FAME, which is a difficult goal for one-dimensional GC [69]. A GCxGC system is composed of 2 columns with stationary phase of different polarities connected to each other by a modulator that periodically collects, focuses, and transfers the sample from the first to the second column. This technique has had applications in very diverse fields such as petrochemical, environment, pharmacy, and food [70, 71].

Lukić et al. used the GCxGC technique coupled to mass spectrometry to analyze virgin olive oils from Croatia, concluding that it is a high efficiency tool, which allows broad determination of volatile compounds of olive oils, which can be associated with the classification of the different varieties, as well as their geographical origin [72].  Table 3 shows the advantages and disadvantages of each previously mentioned chromatographic methods.

## 3. Colorimetric Analysis

### 3.1. ABT Index

The color of oil is an elementary criterion of its quality and fundamental attribute of its organoleptic characteristics that could be a first criterion of the judgment at the time of consuming it [74].

The ABT index is a useful procedure to establish a scale of indices for the denomination of the color of olive oils and seeds, which do not contain reddish hues in view of the human eye; that is, they only represent variable shades from yellow to green. Indicate how many ml of a solution of disodium phosphate there will be in a mixture of said solution with another of monopotassium phosphate, so that, by adding a sufficient number of ml of bromothymol blue, an identical color to that of the oil under study is originated, examining by transparency, with human vision, a 25 mm thick layer of fat, and the standard solution [14].

Tous and Romero performed a color characterization of extra virgin olive oils using this method in Catalonia, Spain, concluding that this method has advantages by having a reduced cost of preparation and ease in handling and interpreting the results obtained [75].

### 3.2. Lovibond Scale

Refined edible oils should have a pale yellow color; such a characteristic can also be measured using the Lovibond tintometer, usually in a range of reds and yellows [20].

This instrumental method is based on the use of techniques such as reflectance or transmittance of the sample that are measured with spectrophotometers or triestimulus colorimeters. The disadvantage of this method is that the surface of the material whose color is to be measured must be homogeneous and measured over a very small area (2 cm^2^), which is not very representative of the result [76].

Karrar et al. used the Lovibond scale to study the effect of heat treatment of Gurum seed oil prior to the extraction process. This index has a direct correlation with the exposure time. The color value was found to increase with an increase in microwave heating at different times [77].

### 3.3. Fritest

It is a colorimetric kit marketed by Merck that detects the amount of carbonyl compounds present in the oil sample. The color of the sample is compared with a scale of 1 (pale yellow) that represents good, 2 (yellow) still good, 3 (yellow-orange) fat to be replaced, and 4 (orange) fat abused [78].

Bansal et al. evaluated the performance of this colorimetric kit when submitting sunflower, sesame, and palm oils to the frying process, in addition to comparing it with other rapid tests such as Oxifritest and Testo 265 [79]. The result was that it is an easy-to-use test to measure the degradation of oil subjected to high temperatures; however, its great limitation is the subjective evaluation of color, which can lead to different results depending on the researcher.

### 3.4. Oxifritest

It is also a colorimetric kit marketed by Merck, but which is based on redox indicators that show the total amount of oxidized compounds in the oil sample [80]. The sample is mixed with two reagents (alcohol and potassium hydroxide) and the color of this mixture is compared with a 4-color scale: blue (1) represents good, blue-green (2) still good, green (3) grease to be replaced, and olive green (4) abused fat.

Bansal et al. concluded that Oxifrit Test has a better use when associated with spectrophotometry since it is subjective in itself, providing a good measure of edible oil deterioration [79].  Table 4 shows the advantages and disadvantages of each previously mentioned colorimetric method.

## 4. Thermoanalytical Methods

The chemical structures present in edible oils determine their thermal stability, with oils with a high proportion of saturated fatty acids being more stable. Being able to perform the thermal analysis of oil has been beneficial to study the physical properties, chemical reactions, and thermal stability of the analyzed oil.

In general, thermoanalytical methods measure the change in weight and enthalpy of the sample when subjected to heating and can also be related to the effect of antioxidant components on the thermal autooxidation of vegetable oils [82]. The most commonly used methods are differential scanning calorimetry (DSC) and thermogravimetric analysis (TGA). The main difference between these techniques is that DSC is a calorimetric method in which energy differences are recorded, while the differences in temperature and weight are recorded in TGA [83].

### 4.1. Thermogravimetric Analysis (TGA)

It is defined as a technique that measures the percentage of lost weight of a sample against time or temperature, while undergoing a temperature controlled program in a specific atmosphere. The atmosphere can be static or dynamic with a given flow rate (reduced pressure conditions are also used), and the most common gases are nitrogen, air, argon, and carbon dioxide [84].

A fundamental characteristic of the TGA technique is that it only allows to detect processes in which there is a variation in weight such as decomposition, sublimation, and absorption, while not allowing to study processes such as mergers and phase transitions. The result of a thermogravimetric analysis is presented graphically, which is known as a thermogram or thermogravimetric curve.

The equipment used in thermogravimetric analysis is a thermobalance that has five main parts:

1. Electronic microbalance and its control equipment
2. An oven and temperature sensors, usually a thermocouple
3. The temperature programmer
4. An atmosphere controller (type of gas and flow).
5. Device for storing weight and temperature data

On the other hand, TGA is able to measure the induction period of edible oil. It is also useful for determining oxidative stability and therefore oil quality [85]. In addition, it is useful to detect the breakdown of fatty acids (PUFA and MUFA) when they are subjected to high temperatures, measuring the formation of hydroxyperoxides and the resistance of fatty acids to oxidation [86].

Flores et al. evaluated the behavior of unrefined Chilean hazelnut oil at high temperatures compared to extra virgin olive oil with the TGA technique, showing that Chilean hazelnut oil can be subjected to high temperatures presenting a good resistance to thermooxidation, obtaining a thermogravimetric curve that shows a three-stage degradation in a range of 100°C to 700°C [87].

### 4.2. Differential Scanning Calorimetry (DSC)

It is a dynamic technique that determines the amount of heat that a substance absorbs or releases when it is at a constant temperature for a certain time, or, when it is heated/cooled at a constant speed over a period of time.

It allows studying processes in which there is an enthalpy variation, establishing the exact moment where physical and/or chemical changes occur, freezing and boiling points, and enthalpy reaction. The advantage of the DSC is that it can be used in wide temperature ranges, from -200°C to approximately 800°C [84].

The DSC uses two capsules: one has the sample to be analyzed, and the other is empty (reference capsule); individual heaters and a control system are used for each capsule as a test if there are temperature differences between the sample and the reference.

The DSC technique allows delivering fusion profiles that are characteristic of each fat matter; it can even be used as a tool to determine the quantitative presence of mixtures of different lipids, since it is associated with changes in the fusion profile, which makes it possible to determine adulteration [88]. Depending on the laboratory conditions, each researcher will decide the most convenient technology to obtain data on the thermal properties of the sample; criteria are summarized in Table 5.

## 5. Methods Used to Determine Antioxidant Activity of Edible Oils

Antioxidants, whether natural or synthetic, are compounds that delay the process of self-oxidation of oil or decrease the rate at which it proceeds, since they inhibit the formation of free radicals while maintaining the quality of food and prolonging its shelf life. In order for them to fulfill this function in oil, natural or synthetic antioxidants must have certain characteristics such as being economical, nontoxic, effective at low concentrations, and stable. Its color, taste, and smell should be minimal in oil [90].

Tocopherols are the natural antioxidants that present, in small quantities, in vegetable oils to ensure the stability of the oil against oxidative deterioration; The most predominant tocopherols in oils are 5,7,8-trimethyl-, 7,8-dimethyl-, and 8-methyltocol [50].

In the food industry, synthetic antioxidants such as butyl hydroxyanisole (BHA), butyl hydroxytoluene (BHT), propyl gallate (PG), ethoxyquin, and terbutyl hydroquinone (TBHQ) are intentionally added [91]. Now, BHA and BHT are the most common phenolic antioxidants, strongly soluble in fats and insoluble in water. BHA is a waxy white solid effective to prevent oxidation of animal fats but ineffective for vegetable fats, while BHT is a fat soluble crystalline solid, suitable for high temperature processing, but not as stable as BHA. It is for this reason that the mixture of them is quite used since they act synergistically providing a greater antioxidant activity in the oil [92].

For frying processes with vegetable oils, TBHQ is more effective than the antioxidants mentioned above and is used together with citric acid, generating excellent synergism in vegetable oils.

On the other hand, ethoxyquin is used in products of animal origin and to avoid superficial burns in fruits. But its use in vegetable oils, despite being effective, should be limited since high concentrations of this antioxidant can cause kidney damage in human and animals [90].

Due to the aforementioned, it is important to be able to determine the antioxidant capacity of oil through different chemical methods, which is useful to study the potential protective effects of the oil and the possible health benefits related to stress-mediated oxidative diseases.

### 5.1. DPPH Method

Among the methods that have been described to evaluate the antioxidant capacity of food and medicinal plants is the technique that uses the free radical 2,2-diphenyl-1-picrylhydrazyl (DPPH). This free radical reacts with compounds that can yield a hydrogen atom, as follows: [93].

(1) $$\begin{array}{c} \left[\text{DPPH}*\right]+\left[\text{AOH}\right]⟶\left[\text{DPPH}‐\text{H}\right]+\left[\text{AO}*\right] \end{array}$$

The method is based on the stability of the DPPH radical, which is attributed to the relocation of the missing electron; this reaction gives it a violet color characterized by an absorption band, in ethanol solution, centered at approximately 520 nm. When the DPPH solution comes into contact with a substance that can yield the hydrogen atom or with another radical, the reduced form DPPH-H or DPPH-R occurs with a loss of color and loss of absorbance [94]. On the other hand, one of the limitations of this method is that although it is a method described more than 50 years ago, the different authors do not use the same concentration of free radical in the reaction media, preventing a precise evaluation of it [95].

Flores et al. evaluated the behavior of Chilean hazelnut oil at high temperatures where DPPH was used for the determination of antioxidant activity, demonstrated the applicability of this method in edible oils, and concluded that at 25°C Chilean hazelnut oil has a better antioxidant activity than olive oil, which decreases at 247°C [87].

### 5.2. ABTS Method

It was developed by Rice-Evans and Miller in 1994 and then modified in 1999; this method consists in the generation of a blue/green ABTS^+^ chromophore through the reaction of ABTS and potassium persulfate. This chromophore is reduced in the presence of hydrogen donor antioxidants measured by spectrophotometry at 734 nm. This method of bleaching is capable of measuring the total antioxidant capacity in lipophilic and hydrophilic substances [93].

Rodriguez et al. evaluated the change in antioxidant activity by the ABTS and DPPH methods during a deep-frying process of a polyunsaturated oil such as sacha inchi oil, indicating that both parameters follow zero-order kinetics. Thus, a polyunsaturated oil such as the one studied is suitable for short-term deep-frying of french fries [96].

### 5.3. Oxygen Radical Absorbance Capacity (ORAC) Method

It measures the radical breaking capacity of the antioxidant chain through the control of peroxyl radical inhibition. Peroxyl radicals are found in the oxidation of food lipids and biological systems under physiological conditions; the following reaction occurs:

(2) $$\begin{array}{c} \text{R}‐\text{N}=\text{N}‐\text{R}+2{\text{O}}_{2}⟶{\text{N}}_{2}+2\cdot \text{ROO}\cdot \end{array}$$

Following this, ORAC values are considered, by some scientists, relevant at the biological level to show antioxidant efficacy. In this test, the peroxyl radical that is produced reacts with a fluorescent probe resulting in the loss of fluorescence, which is recorded with a fluorimeter. Therefore, a set of fluorescence decomposition curves can be constructed in the absence or presence of antioxidants. As standardization, a standard antioxidant is used, usually Trolox, and the ORAC values of the tested antioxidants are reported as equivalent to Trolox. ORAC measures the donation capacity of the hydrogen atom of antioxidants. This procedure is easily automated using multichannel liquid handling together with a fluorescence microplate reader [92]. Ou et al. when applying this method in edible oils concluded that, despite being effective, reliable, and reproducible, it has the limitation that it depends on the reagents that are used since, if they are photosensitive, it will affect the sample [97]. Despite the robustness of the method, not all studies show homogeneity of results and must be modified in order to be used [98].

### 5.4. Ferric Reducing Antioxidant Power (FRAP) Method

Technique initially developed by Benzie and Strain (1996) to measure the reducing power in plasma, but later modified to study antioxidants in botanical; directly measuring the reduction of the Fe^+3^ 2,4,6-tripyridyl-s-triazine complex (TPTZ) to the Fe^+2^ complex of intense blue color by the antioxidants in an acid medium. Results are obtained as absorbance is increased to 593 nm in the spectrophotometer [98, 99].

This method is simple and fast. However, the main limitation of this type of assay is that it can only provide the total antioxidant potential of the sample, without information on which specific components could be responsible for the antioxidant behavior [100, 101]. On the other hand, according to Kaseke et al. temperature, pressure, and extraction time are critical factors to determine the antioxidant capacity of seed oil from subcritical and supercritical fluid extractions, having an increasing the temperature to 60°C and reducing the pressure to 2 MPa a negative effect for the FRAP test for edible fruit seed oils [102]. This method was used to evaluate the antioxidant activity of extra virgin olive oil being compared with DPPH and ABTS, concluding that the oil metabolite composition will determine the antioxidant capacity and also which method is optimal to use [103]. A summary can be seen in Table 6 of the characteristics of the different methods mentioned previously.

## 6. Conclusions

Monitoring the deterioration of a real fat is a complex procedure to carry out; it is a study of components that are found in different proportions in the lipid matrix and that also have a different behavior and that can become more complex even if there is participation of exogenous agents as in the frying process.

The different methodologies published in the literature determine the concentration of the nutritional compounds of the fat, as well as the compounds that are generated as a result of the degradation of the different original compounds, the so-called primary and secondary oxidation compounds, in order to provide a complete understanding of lipid impairment.

During the development of this review, it has been highlighted how a wide set of analytical methods and techniques has been used, ranging from classical volumetry (FFA, PV) to other less common techniques (triacylglycerol composition, differential scanning calorimetry, and thermogravimetric analysis), without excluding chromatographic techniques, spectroscopy, spectrometry, and the use of electrical conductivity sensors.

In this sense, despite the fact that this review focuses on the chemical and physical parameters of edible fats and oils, the possible applications go beyond fats for human consumption, possibly including other materials of industrial interest. Different methods have different purposes, although the use of basic methods can be applied advantageously when the analyzed matrix is well known, sophisticated methods allow us to determine the compositional changes suffered by the majority and minority components of lipids during a deterioration process with greater specificity, precision, and accuracy, as well as the determination of the oxidative stability of the different fats, especially when it comes to an unknown lipid material. Finally, despite having a substantial number of method and technology studies, there is the challenge of improving the times involved mainly with sophisticated methodologies, as well as improving the access of these latest technologies to countries with less developed economies.

## Figures and Tables

**Table 1:** Advantages and disadvantages of the basic characterization methods of fat and oils.

Name	Principle to use	Instrumentation	Advantages	Disadvantages	Ref.
Acid value	Acid base reaction	Titration using burette	Reproducible, low cost, reliable results	Depends on human vision, based on only one fatty acid	[6, 15]
Iodine value	Oxide reduction reaction	Titration using burette	Reproducible, low cost, reliable results	Excess titration, indirect procedure	[6, 22]
Saponification value	Saponification reaction	Titration using burette	Reproducible, low cost, reliable results	Dependent on temperature and human vision	[6, 19]
Thiobarbituric acid index	Formation of the alkylidentiobarbituric acids	Spectrophotometer	Good correlation with other chemical methods and sensory evaluation	Time consuming	[23, 24]
Total polar compounds (TPC)	Adsorption	Column chromatography	This method is accurate and reproducible. Oils with similar PUFA good correlation between TPC and degradation products. Kinetics parameters	Time consuming, weak correlation between TPC and oils with a high content of diacylglycerols	[25, 26]

**Table 2:** Advantages and disadvantages of the methods of accelerated oxidation conditions.

Name	Principle to use	Instrumentation	Advantages	Disadvantages	Ref.
Rancimat	Electric conductivity	Rancimat equipment	Does not use reagents, reproducible	Indirect measure of lipid deterioration	[46]
Active oxygen	Oil rancidification	Glass tubes	Reproducible, used for more than 60 years	It depends on the stability of the peroxides generated.	[20, 49]
Schaal stove test	Heating, convection	Oven	Reproducible, reliable and economical	It only measures the final result of the study.	[56]
Microwave	Electromagnetic waves	Oven	Fast, uniform, economical	Does not provide analytical measurements	[57]

**Table 3:** Advantages and disadvantages of chromatographic methods.

Name	Principle to use	Instrumentation	Advantages	Disadvantages	Ref.
Column chromatography	Adsorption	Chromatography column	Easy to handle and widely used	Multi-stage process so it is slow.	[73]
Gas chromatography	Compound separation	Chromatograph fitted with detectors such as FID or MS	Effective for studying compounds with high boiling points	It needs derivatization processes, in addition to time consuming and high cost equipment	[66]
Two-dimensional gas chromatography	Compound separation	Chromatography columns	High efficiency, in addition to analyzing aromatic compounds	It needs derivatization processes, in addition to high cost equipment	[72]

**Table 4:** Advantages and disadvantages of colorimetric methods.

Name	Principle to use	Instrumentation	Advantages	Disadvantages	References
ABT index	Transparency	Human vision	Low cost of preparation and easy handling	Exclusive use of olive oil and seeds	[75]
Lovibond scale	Reflectance or transmittance.	Spectrophotometer or colorimeter.	Easy use and low price	Use in refined oils	[81]
Fritest	Comparison with color scale	Human vision	Low price and simple manipulation	Subjective evaluation of oil color	[78]
Oxifritest	Comparison of oils with colorimetric scale	Human vision	Reflects oxidized compounds	Subjective evaluation of oil color	[79, 80]

**Table 5:** Advantages and disadvantages of thermogravimetric methods.

Name	Principle to use	Instrumentation	Advantages	Disadvantages	Applications
TGA	Thermogravimetry	Thermobalance	It allows weight loss to be associated with degradation of polar compounds in dynamic processes	The residual sample cannot be measured since it burns completely	Thermal behavior of vegetable oils. [86, 87]
DSC	Calorimetry	Capsules and heaters	It allows to study process in which there is enthalpy variation	It requires researcher experience for the correct analysis	Evaluation of thermal properties, kinetic parameters [89].

**Table 6:** Advantages and disadvantages of the methods to measure antioxidant capacity.

Name	DPPH method	ABTS method	ORAC method	FRAP method
Radical or active species	Free radical 2,2-diphenyl-1-picrylhydrazyl.	Azinobis 3-ethylbenzothiazoline-6-sulfonic acid)	Peroxyl radicals	Fe^+3^ 2,4,6-tripyridyl-s-triazine (TPTZ)
Equipment	Spectrophotometer	Spectrophotometer	Fluorometer	Spectrophotometer
Advantage	Quick preparation	Detects hydrophilic and lipophilic antioxidants	It can detect antioxidant activity widely	Higher absorbance spectrum than the other methods
Disadvantages	The results vary according to the concentration of the free radical.	It is not a physiological radical, so it requires more preparation time	It must be used in in vitro samples	It is less accurate than the other methods, since the reducing power is not always antioxidant capacity.
Applications	Antioxidant activity in unrefined oils and native species. [87, 95].	Food industry	Food industry, biological fluids [97]	Food industry, botany, medicinal [104]

## Data Availability

The data supporting this systematic review are from previously reported studies and datasets, which have been cited.
